# Colorectal submucosal invasive carcinoma with a small mucinous component at the invasive front resulting in heterochronic metastasis

**DOI:** 10.1016/j.igie.2023.07.006

**Published:** 2023-07-15

**Authors:** Yui Tanaka, Ryo Seishima, Yutaka Kurebayashi, Shimpei Matsui, Kohei Shigeta, Koji Okabayashi, Yuko Kitagawa

**Affiliations:** 1 Department of Surgery, Keio University School of Medicine, Tokyo, Japan; 2 Department of Pathology, Keio University School of Medicine, Tokyo, Japan

A 75-year-old women visited our hospital after she had a positive result on a fecal occult blood test. Colonoscopy revealed a laterally spreading 85 mm tumor in the lower rectum (Fig. 1). Via magnifying endoscopy with crystal violet staining, an irregular pit pattern was visualized (Kudo’s classification type V), and partial submucosal invasion was suspected (Fig. 2). The patient was informed of the risk of recurrence if treated endoscopically and that surgery was recommended. However, in accordance with the patient’s wishes, endoscopic submucosal dissection was performed instead of surgical resection (Figs. 3 and 4). Results of pathologic examination revealed a predominantly well-differentiated adenocarcinoma with a small mucinous carcinoma component at the apical invasive front (Figs. 5 and 6). The tumor size was 85 × 75 mm, and there was no lymphovascular invasion. All resection margins were negative, but the tumor had invaded up to 1500 μm of the submucosa and was indicated for surgical resection. However, the patient chose to undergo strict follow-up. Colonoscopy was performed once a year for 3 years after endoscopic submucosal dissection and once every 3 years thereafter. In addition, contrast-enhanced CT imaging was performed once a year.

**Figure 1:**
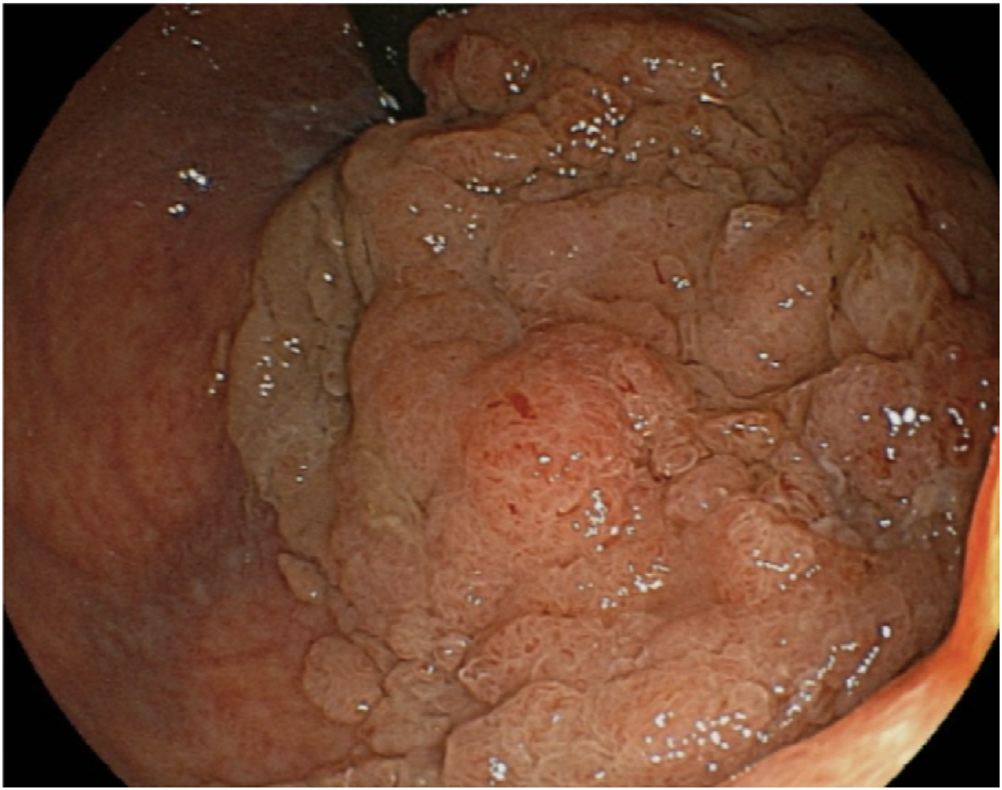
Colonoscopy revealed a laterally spreading tumor in the lower rectum.

**Figure 2:**
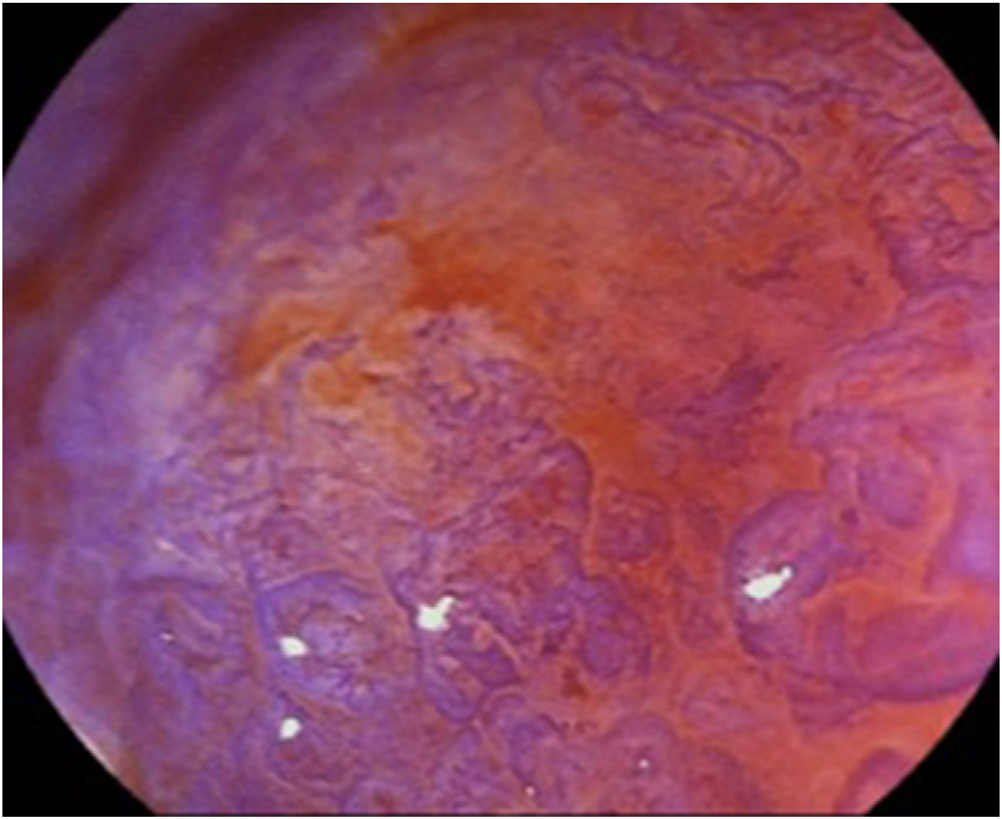
Magnifying endoscopy with crystal violet staining revealed an irregular pit pattern (Kudo’s classification type V).

**Figure 3:**
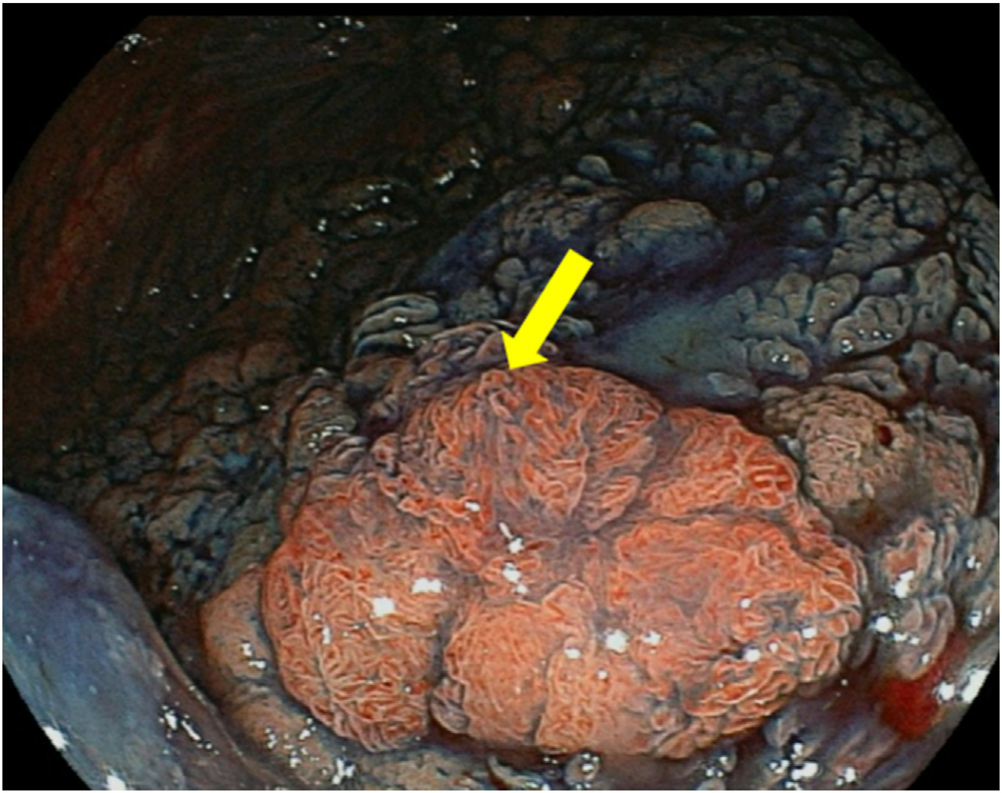
Colonoscopy image of the tumor. The *yellow arrow* indicates the pathologically confirmed area of the mucinous component.

**Figure 4:**
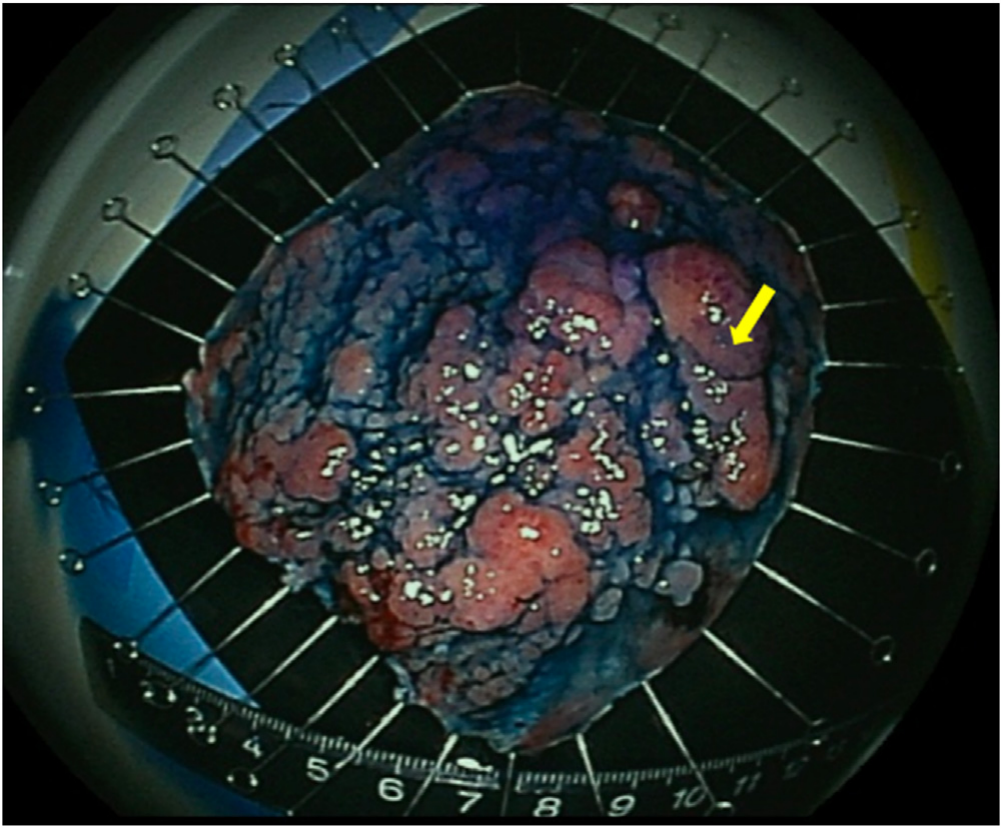
Image of the resected specimen after endoscopic submucosal dissection. The *yellow arrow* shows the pathologically confirmed area of the mucinous component.

**Figure 5:**
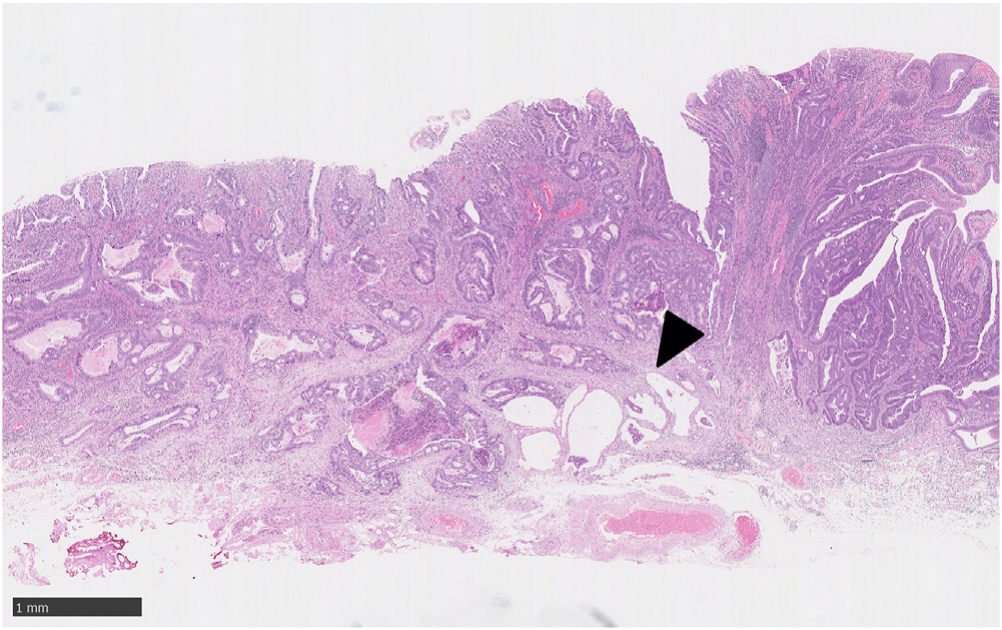
Low magnification image of the resected specimen. A predominant, well-differentiated adenocarcinoma with a small mucinous carcinoma component at the apical invasive front (*arrowhead*) was observed (H&E, orig. mag. ×40).

**Figure 6:**
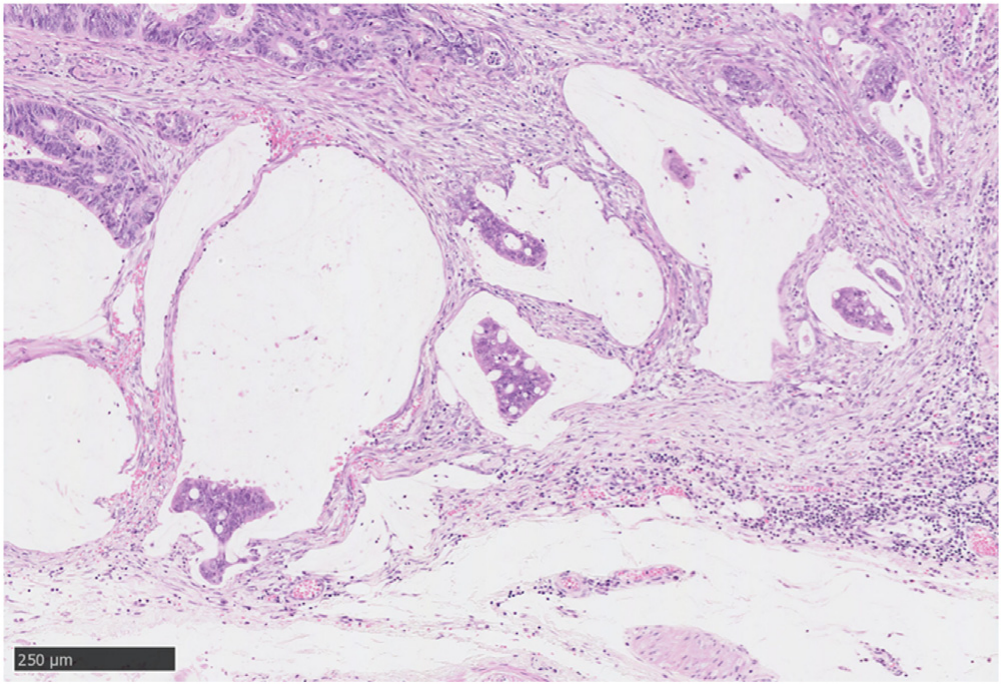
The higher power field image of the *arrowhead* in Figure 5 (H&E, orig. mag. ×100).

Ten years later, a rapidly growing 3-cm-sized suspected lymph node recurrence was found within the mesorectum on the left side by contrast-enhanced CT imaging and positron emission tomography/CT imaging (Fig. 7). The patient agreed to undergo surgery, and laparoscopic low anterior resection was performed. The pathology showed lymph node metastasis of mucinous carcinoma, which was consistent with metastasis from rectal cancer (Fig. 8).

**Figure 7:**
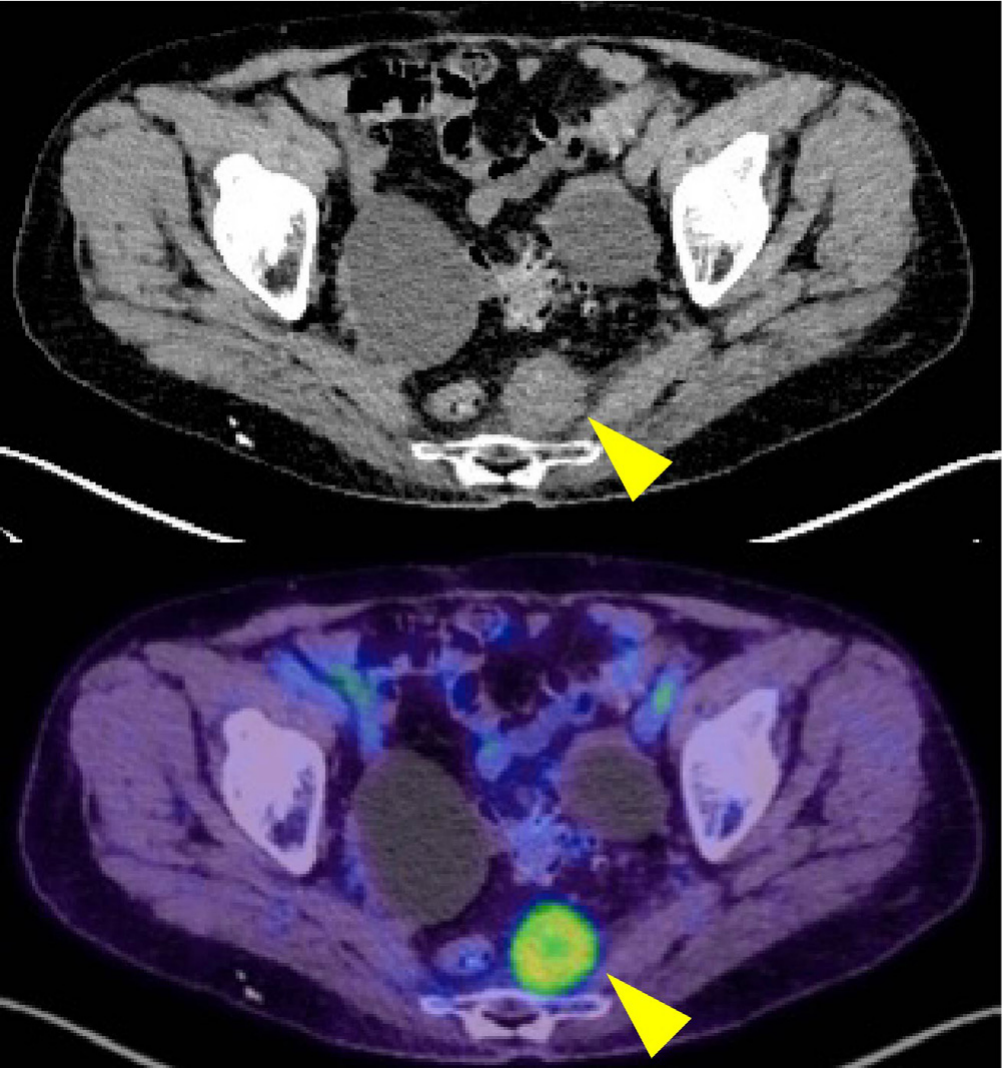
Contrast-enhanced CT and positron emission tomography/CT images. A 3-cm-sized lymph node within the mesorectum (*arrowhead*) was found on the left side.

**Figure 8:**
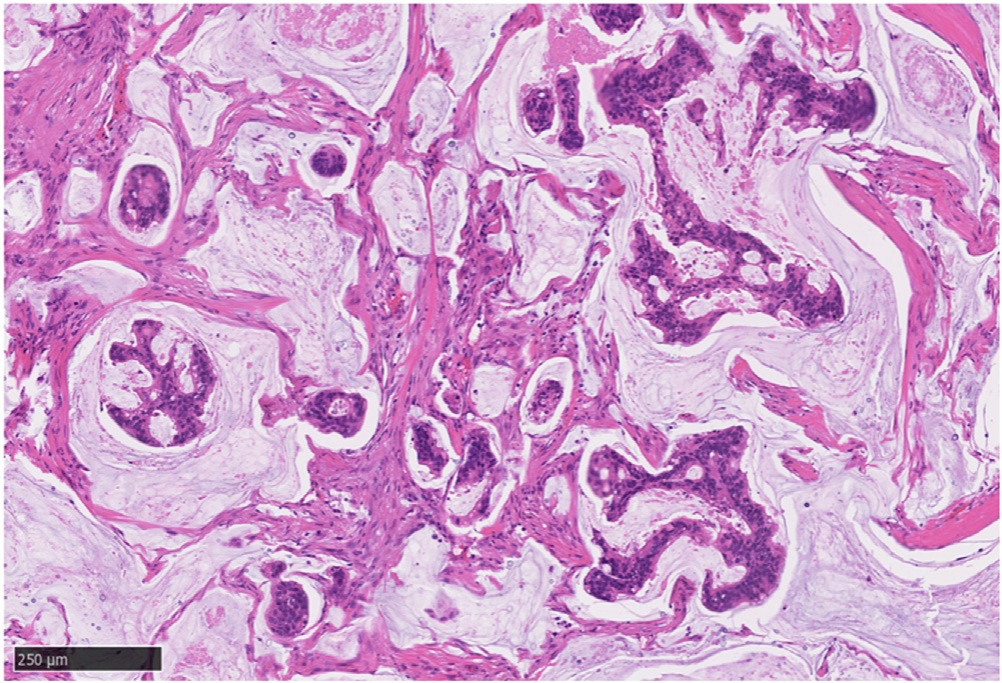
Atypical epithelial cells with dense nuclear staining and large, atypical nuclei proliferate in a vesicular to tubular fashion, floating in a mucus lake (H&E, orig. mag. ×100).

Small amounts of mucinous carcinoma components are often found mixed with predominantly differentiated adenocarcinomas. It has been reported that patients with mucinous carcinoma components at the apical invasive front have a poor prognosis, but the need for subsequent surgical resection remains controversial. Furthermore, no previous cases have been reported in which such cancer cells have been proven to cause heterochronic recurrence. The current case clearly shows evidence for such a recurrence. Future discussions should address the indication for surgical resection and the appropriate surveillance interval for such cases.

## Disclosure

All authors disclosed no financial relationships.

